# Therapeutic Targeting of Apoptosis, Autophagic Cell Death, Necroptosis, Pyroptosis, and Ferroptosis Pathways in Oral Squamous Cell Carcinoma: Molecular Mechanisms and Potential Strategies

**DOI:** 10.3390/biomedicines13071745

**Published:** 2025-07-16

**Authors:** Po-Chih Hsu, Chung-Che Tsai, Ya-Hsuan Lin, Chan-Yen Kuo

**Affiliations:** 1 Department of Dentistry, Taipei Tzu Chi Hospital, The Buddhist Tzu Chi Medical Foundation, New Taipei City 231, Taiwan; pchsu@gms.tcu.edu.tw; 2 Institute of Oral Medicine and Materials, College of Medicine, Tzu Chi University, Hualien 970, Taiwan; 3 Department of Research, Taipei Tzu Chi Hospital, The Buddhist Tzu Chi Medical Foundation, New Taipei City 231, Taiwan; chungche.tsai@gmail.com; 4 Department of Nursing, Cardinal Tien College of Healthcare and Management, New Taipei City 231, Taiwan; 5 Department of Chinese Medicine, Taipei Tzu Chi Hospital, The Buddhist Tzu Chi Medical Foundation, New Taipei City 231, Taiwan; iamacer39@tzuchi.com.tw

**Keywords:** oral squamous cell carcinoma (OSCC), regulated cell death, apoptosis, autophagic cell death, necroptosis, pyroptosis, ferroptosis

## Abstract

Oral squamous cell carcinoma (OSCC) is a prevalent and aggressive malignancy with poor prognosis, largely due to its high metastatic potential and resistance to conventional therapies. Recent advances in cancer biology have underscored the significance of regulated cell death pathways, including apoptosis, autophagic cell death (ACD), necroptosis, pyroptosis, and ferroptosis, in modulating tumor progression and therapeutic responses. This review provides the current insights into the molecular mechanisms underlying these cell death pathways and explores their therapeutic relevance in OSCC. Restoration of apoptosis using BH3 mimetics, tumor necrosis factor (TNF)-related apoptosis-inducing ligand (TRAIL) receptor agonists, and p53 reactivators shows promise for sensitizing OSCC cells to treatment. Autophagy plays context-dependent roles in cancer, acting as a tumor suppressor during early carcinogenesis by maintaining cellular homeostasis, and as a tumor promoter in established tumors by supporting cancer cell survival under stress. Targeting necroptosis and pyroptosis has emerged as a novel strategy for inducing cancer cell death, with compounds such as acetylshikonin and okanin demonstrating antitumor effects. Additionally, the induction of ferroptosis via lipid peroxidation and glutathione peroxidase 4 (GPX4) inhibition offers a promising avenue for overcoming drug resistance, with agents such as quercetin and trifluoperazine exhibiting preclinical success. Integration of these therapeutic approaches may enhance the OSCC treatment efficacy, reduce chemoresistance, and provide novel prognostic biomarkers for clinical management. Future studies should focus on optimizing combinatorial strategies that effectively leverage these pathways to improve OSCC patient outcomes.

## 1. Introduction

Oral cancer accounts for approximately 48% of all head and neck malignancies, and over 90% of these cases are histologically confirmed as oral squamous cell carcinoma (OSCC) [1,2]. The risk factors include tobacco use, alcohol consumption, human papillomavirus (HPV) infection, and genetic predisposition [3]. Understanding the diverse modes of cell death is essential for developing targeted treatments and overcoming resistance to therapy [4]. Recently, the complexity and significance of various cell death pathways have gained attention in the context of tumor biology [5]. Cancer cells often acquire the ability to evade programmed cell death, which contributes to uncontrolled proliferation, metastasis, and therapeutic failure. Traditional treatments such as surgery, radiotherapy, and chemotherapy may be limited by intrinsic or acquired resistance that stems from altered cell death signaling [6]. Hence, elucidating the molecular basis of different cell death modalities in OSCC is critical not only for understanding disease pathogenesis but also for discovering novel and effective treatment options [7].

A comprehensive analysis of cell death mechanisms in OSCC has revealed a highly intricate and interwoven network of pathways. These include traditional forms of programmed cell death such as apoptosis, autophagic cell death (ACD), and necrosis, which have long been studied in the context of tumor progression and therapeutic responses [8]. Recently, attention has turned to newly characterized mechanisms such as ferroptosis, pyroptosis, and necroptosis, which exhibit unique molecular triggers and morphological features [9]. Additionally, emerging forms of cell death, such as cuproptosis (copper-induced death), anoikis (detachment-induced apoptosis), parthanatos (PARP1-dependent cell death), and entosis (cell-in-cell invasion leading to death), are gaining relevance in cancer biology [7,10,11]. Entosis is a cellular event in which a cell integrates into another cell. There are some potential events after becoming entosis. Cell death is only a possibility after entosis. In other words, cells could survive after entosis, or the entered cell could exit from the recipient cell without cell death [12]. These pathways not only add layers of complexity to the understanding of tumor cell fate but also provide novel therapeutic opportunities to overcome resistance and improve patient outcomes [9]. These mechanisms are interconnected and can influence one another, creating a dynamic network that determines cell fate [13]. Advances in molecular oncology have revealed that targeting specific nodes within these pathways can sensitize cancer cells to therapies and stimulate antitumor immunity. This review presents a comprehensive analysis of the role that various cell death pathways play in oral cancer. Additionally, it evaluates therapeutic strategies designed to manipulate these pathways for potential clinical advantage.

## 2. Targeting Apoptotic Pathways in OSCC: Mechanisms and Therapeutic Strategies

**Figure 1:**
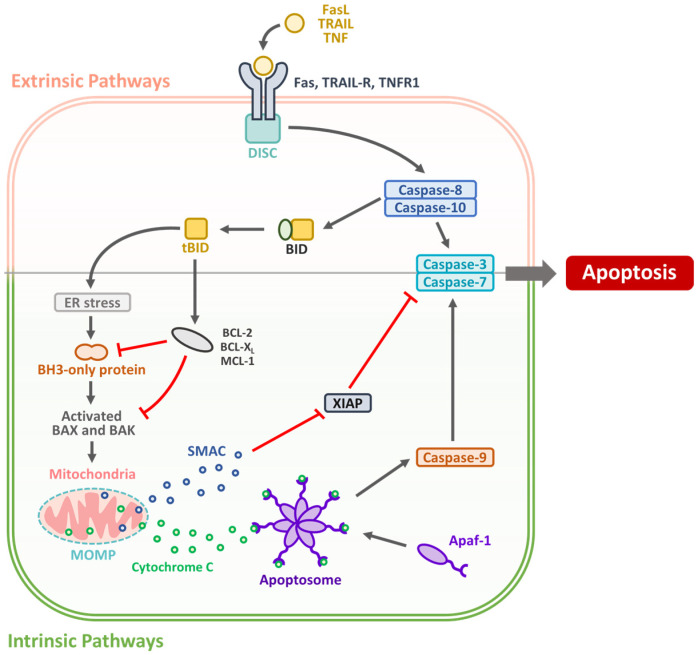
Molecular cascades of extrinsic and intrinsic apoptosis pathways. The extrinsic pathway is triggered by death ligands, including Fas ligand (FasL), TRAIL, and TNF, which bind to their respective receptors (Fas, TRAIL-R, and TNFR1). This leads to the formation of the death-inducing signaling complex (DISC) and the activation of cysteine-dependent aspartate-specific proteases (caspases), caspase-8/10, which then activate caspase-3/7. Caspase-8 also cleaves BH3 interacting-domain death agonist (BID) to tBID, linking to the intrinsic pathway. Intrinsic signals, including endoplasmic reticulum (ER) stress, activate BAX/BAK, inducing mitochondrial outer membrane permeabilization (MOMP), cytochrome c and SMAC release, apoptosome formation (via APAF1), and caspase-9 activation. XIAP inhibits caspases, while SMAC neutralizes XIAP.

Apoptosis, or programmed cell death, is an essential physiological process that maintains tissue homeostasis by eliminating damaged or unwanted cells without inducing inflammation. In OSCC, evasion of apoptosis is a hallmark of tumor progression and treatment resistance [14]. Apoptotic signaling occurs primarily through two interconnected pathways: the intrinsic (mitochondrial) and extrinsic (death receptor) pathways, both of which are often dysregulated in OSCC [15]. The dysregulation of these pathways plays a crucial role in OSCC carcinogenesis, progression, and resistance to therapy [16]. The intrinsic pathway is often disrupted by the overexpression of anti-apoptotic B-cell lymphoma 2 (Bcl-2) family members, including Bcl-2 and B-cell lymphoma-extra large (Bcl-xL), and downregulation or mutation of pro-apoptotic proteins, such as Bcl2-associated X protein (Bax), Bcl-2 homologous antagonist/killer (Bak), and p53 [17]. Mutations in tumor protein p53 (*TP53*) are among the most frequent genetic alterations in OSCC and lead to impaired DNA damage responses and apoptotic signaling [18]. The extrinsic pathway involves death receptors (DRs), such as the Fas cell surface death receptor (Fas), tumor necrosis factor (TNF)-receptor I (TNFR1), and TNF-related apoptosis-inducing ligand (TRAIL) receptor (TRAIL-R), which can be downregulated or rendered nonfunctional in OSCC cells, further promoting survival and resistance to immune-mediated apoptosis [19]. The apoptotic mechanism is illustrated in Figure 1.

Inhibitors of apoptosis proteins (IAPs), including x-linked IAP (XIAP) and survivin, are upregulated in OSCC and contribute to apoptotic resistance [20]. Their expression is associated with poor prognosis, aggressive tumor behavior, and reduced sensitivity to chemotherapy and radiotherapy [21]. Several therapeutic strategies have been developed to restore apoptosis in OSCC [22]. BH3 mimetics such as ABT-199 (Venetoclax) target Bcl-2 proteins to activate intrinsic pathways [23]. Reactivation of p53 through small molecules such as PRIMA-1 or APR-246 is another promising strategy [24]. Death receptor agonists and TRAIL-based therapies aim to engage the extrinsic pathway, although their pre-clinical translation has faced challenges due to tumor heterogeneity and resistance mechanisms [25]. IAP antagonists, such as second mitochondria-derived activator of caspase (SMAC) mimetics, have also been investigated to sensitize tumor cells to apoptosis-inducing treatments [26]. However, targeting apoptosis holds significant promise for improving treatment efficacy in OSCC, particularly in combination with conventional therapies or immune checkpoint inhibitors [27]. Ongoing research aims to identify predictive biomarkers for apoptotic response and develop combination regimens that overcome resistance and enhance tumor cell eradication [28]. Restoring apoptotic signaling through targeted therapies holds significant promise for OSCC treatment. Combining apoptosis-inducing agents with conventional therapies or immune checkpoint inhibitors may enhance treatment efficacy, reduce drug resistance, and improve patient outcomes (Table 1).

## 3. Therapeutic Targeting of ACD in OSCC: Dual Roles, Regulatory Pathways, and Emerging Strategies

**Figure 2:**
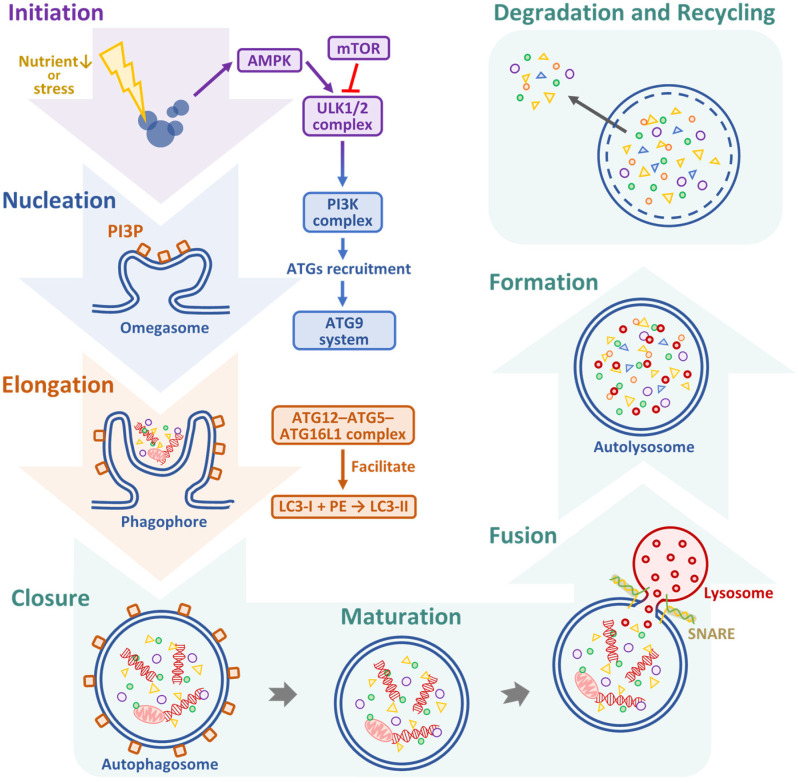
Mechanistic overview of the autophagic process. Autophagy involves sequential progression through initiation, nucleation, elongation, maturation, fusion, and degradation. The pathway is activated by AMPK and inhibited by mTOR. During autophagy, cellular macromolecules are enclosed within double-membrane vesicles called autophagosomes, whose formation is orchestrated by multiprotein ATG complexes that remodel membranes at each stage. Once formed, autophagosomes fuse with lysosomes to create autolysosomes, where their contents are degraded and recycled.

Autophagy is a highly conserved lysosomal degradation process that maintains cellular homeostasis by removing damaged organelles, misfolded proteins, and other intracellular debris [29]. In OSCC, autophagy plays a critical role, functioning as both a tumor suppressor in the early stages of carcinogenesis and a tumor promoter in the advanced stages [30]. Therapeutic modulation of autophagy can yield opposing effects depending on disease stage, and autophagy activation may prevent tumor initiation, while autophagy inhibition can enhance treatment efficacy in advanced cancers [31]. Autophagy proceeds through a series of coordinated steps, including initiation, nucleation, elongation, autophagosome formation, fusion, and degradation [32]. Autophagy initiation is tightly regulated by nutrient and energy-sensing signaling pathways, primarily the phosphoinositide 3-kinase (PI3K)/protein kinase B (AKT)/mammalian target of rapamycin (mTOR) and AMP-activated protein kinase (AMPK) pathways [33]. Under nutrient-rich conditions, mTOR inhibits autophagy by phosphorylating and inactivating the Unc-51-like kinase 1 (ULK1) complex. In contrast, energy depletion activates AMPK, which directly phosphorylates and activates ULK1 while simultaneously inhibiting mTOR, thereby promoting autophagy initiation [34,35]. Following ULK1 activation, the class III PI3K complex comprising vacuolar protein sorting 34 (VPS34), Beclin-1 (BECN1), VPS15, and autophagy-related (ATG) 14 is recruited to the phagophore assembly site, where it generates phosphatidylinositol 3-phosphate, a critical lipid signal for attracting downstream ATG proteins [36]. Among these components, BECN1 plays a central regulatory role in autophagosome nucleation [37]. Its expression and function are frequently altered in OSCC and other malignancies, leading to impaired autophagosome formation and contributing to tumor progression and altered cell survival dynamics. During the elongation and maturation of the phagophore membrane, two ubiquitin-like conjugation systems are essential for autophagosome formation. The first involves the conjugation of ATG12 to ATG5, which then forms a complex with ATG16L1 to facilitate membrane expansion [38]. The second system is responsible for the lipidation of microtubule-associated protein 1 light chain 3 (LC3). In this process, cytosolic LC3-I is conjugated to phosphatidylethanolamine (PE) to form LC3-II, which is incorporated into the autophagosome membrane and serves as a widely used marker of autophagic activity [39]. These molecular events are tightly coordinated and necessary for the proper assembly and closure of the double-membrane autophagosome structure. In addition, autophagy adaptor proteins such as sequestosome 1 (SQSTM1, also known as p62) play an important role by linking ubiquitinated cargo to LC3-II, thereby ensuring selective degradation of protein aggregates and damaged organelles. Dysregulation of these processes, including altered LC3 lipidation or p62 accumulation, has been implicated in various cancers where they influence tumor cell survival, therapeutic resistance, and disease progression [40]. In the step of fusion and degradation, once the autophagosome is fully formed, it undergoes fusion with lysosomes to generate autolysosomes, wherein the inner membrane and its enclosed cargo are degraded by lysosomal hydrolases [41]. This critical step is orchestrated by several key molecular mediators, including soluble N-ethylmaleimide-sensitive factor activating protein receptor (SNARE) proteins, the small guanosine triphosphatase (GTPase) Ras-related protein 7 (Rab7), and the homotypic fusion and vacuolar protein sorting (HOPS) tethering complex, all of which coordinate vesicle docking and membrane fusion [42]. The breakdown of cargo within the autolysosome releases essential metabolites such as amino acids, lipids, and sugars, which are subsequently recycled to support cellular energy balance and biosynthetic processes, particularly during periods of metabolic stress or nutrient deprivation [43]. The autophagic pathway can be mechanistically categorized into seven major phases, as represented in Figure 2.

Autophagy, initially tumor-suppressive, often shifts to a pro-survival role in advanced cancers, aiding cells to cope with stressful conditions [44]. Reactive oxygen species (ROS) both induce and result from autophagy, forming a feedback loop that influences tumor progression. Key pathways such as PI3K/AKT/mTOR and AMPK regulate this axis and present therapeutic targets [45]. The key regulatory proteins involved in autophagy include BECN1, LC3, and p62. Loss of BECN1 or downregulation of LC3 has been correlated with OSCC progression, highlighting the tumor-suppressive role of autophagy [19]. NK2 homeobox 3 (NKX2-3), Fas-associated death domain protein (FADD), parkin RBR E3 ubiquitin protein ligase (PARK2), and epidermal growth factor receptor (EGFR) have been identified as potential prognostic markers associated with autophagy in the tumorigenesis of head and neck squamous cell carcinomas [46,47]. Autophagy, a cellular degradation process, has been implicated in OSCC, but its precise role in cancer progression remains unclear, as its regulation appears to be both time- and cell-dependent [48]. Additionally, in established OSCC tumors, cancer cells may exploit autophagy as a survival mechanism to endure metabolic stress, hypoxia, and nutrient deprivation [48]. Increased autophagic flux can confer resistance to chemotherapy and radiotherapy because cancer cells utilize autophagy to recycle intracellular components and sustain energy production [49] (Table 2).

Therapeutic strategies targeting ACD in OSCC are currently under-investigated [50]. The combination of NaAsO_2_, CQ, and dichloroacetate (DCA) is a promising therapeutic strategy for overcoming chemoresistance in OSCC by concurrently targeting oxidative stress, ACD, and glycolysis in vivo [51]. Apicidin is a histone deacetylase (HDAC) inhibitor that effectively induces apoptosis and ACD in OSCC cells, and inhibition of ACD enhances its pro-apoptotic effects [52]. A study explores the important role of ACD in OSCC using a 4NQO-induced carcinogenesis model. Treatment with spermidine (SPD) reduces lesion severity and incidence of OSCC by inducing ACD, decreasing DNA damage, and reducing oxidative stress. Conversely, ACD inhibition with chloroquine provided no protective effects. Plasma C16 ceramide levels correlated with lesion severity and were lower in SPD-treated mice, suggesting their potential as biomarkers [53]. Resveratrol effectively induces both ACD and apoptosis in cisplatin-resistant oral cancer cells through AMPK activation and autophagic gene upregulation [54]. In cisplatin-resistant chimeric antigen receptor (CAR) cells, resveratrol treatment significantly increased the protein levels of caspase-3 and -9, cytochrome c, apoptotic protease-activating factor-1 (Apaf-1), apoptosis-inducing factor (AIF), endonuclease G (Endo G), Bax, and Bad, while decreasing Bcl-2 expression and phosphorylation of Bad at Ser136, indicating that apoptosis is triggered via a mitochondria-dependent pathway alongside ACD induction [54]. Han et al. reported that piperine triggers apoptosis and autophagy in oral cancer cells by inhibiting the PI3K/AKT/mTOR pathway in vitro, and exhibits significant antitumor activity in vivo [55]. Bortezomib enhances radiosensitivity in OSCC by promoting the autophagic degradation of tumor necrosis factor receptor-associated factor 6 (TRAF6), leading to reduced tumor growth and survival in vitro and in vivo [56]. Therefore, targeting ACD is a promising therapeutic strategy for OSCC, and several natural compounds and inhibitors have demonstrated the potential to enhance apoptosis, reduce tumor growth, and overcome chemoresistance (Table 3).

## 4. Necroptosis in OSCC: Mechanisms, Molecular Markers, and Therapeutic Implications

**Figure 3:**
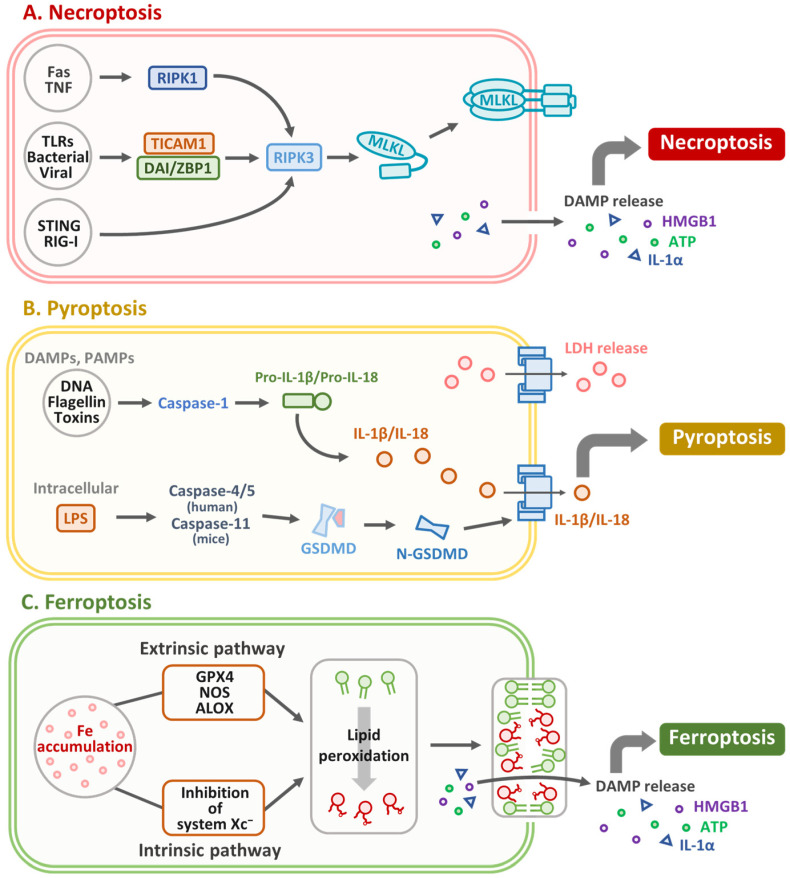
Molecular mechanisms of necroptosis, pyroptosis, and ferroptosis: (**A**) Necroptosis is a regulated form of necrotic cell death triggered by DRs (Fas and TNF), TLRs, or cytosolic sensors such as stimulator of interferon genes (STING) and retinoic acid-inducible gene I (RIG-I). These signals activate RIPK1, TIR domain containing adaptor molecule 1 (TICAM1), or Z-DNA binding protein 1 (ZBP1), which converge on RIPK3 to phosphorylate MLKL. This leads to membrane rupture and release of damage-associated molecular patterns (DAMPs), including high mobility group box 1 (HMGB1), adenosine triphosphate (ATP), and interleukin (IL)-1α. (**B**) Pyroptosis is mediated by inflammatory caspases. Caspase-1, activated by DAMPs or PAMPs, cleaves pro-IL-1β/IL-18 and gasdermin D (GSDMD). Lipopolysaccharide (LPS) activates caspase-4/5 (human) or caspase-11 (mouse), also targeting GSDMD. The N-terminal GSDMD fragment forms membrane pores, leading to release of IL-1β, IL-18, and lactate dehydrogenase (LDH). (**C**) Ferroptosis is an iron-dependent form of cell death driven by lipid peroxidation. It is induced by iron accumulation, glutathione peroxidase 4 (GPX4) inhibition, or suppression of system Xc^−^, leading to glutathione depletion and ROS accumulation, which compromise membrane integrity and trigger ferroptotic death with DAMP release.

Necroptosis was first identified as a type of programmed cell death by Degterev et al. in 2005 [57]. It is a distinct, regulated form of programmed cell death characterized by specific morphological and biochemical features, such as plasma membrane rupture, organelle swelling, cytoplasmic and nuclear disintegration, leakage of cellular contents, and release of damage-associated molecular patterns, triggering inflammatory responses [58]. Although necroptosis resembles necrosis morphologically, it differs fundamentally in that necrosis is a passive, unregulated response to overwhelming cellular damage, whereas necroptosis is a genetically controlled process mediated by receptor-interacting protein kinases. Notably, necroptosis can act as both a detrimental and protective mechanism, promoting inflammation, tissue damage, and tumor progression in some contexts, while exerting antiviral, antibacterial, and antitumor effects in others. The necroptotic pathway is mediated by key molecules such as receptor-interacting serine/threonine-protein kinase 1 (RIPK1), RIPK3, and mixed lineage kinase domain-like pseudokinase (MLKL) (Figure 3A). It can be activated by DRs such as TNFR1 and Toll-like receptors (TLRs) [59,60]. Tumor necrosis and necroptosis are common histopathological features of solid tumors, including OSCC [61]. Recent clinical–cohort analyses have identified a necroptosis-related gene signature that stratifies OSCC patients into high- and low-risk groups with significantly different survival outcomes [62]. Comprehensive reviews have further elucidated the dual roles of RIPK1, RIPK3, and MLKL in OSCC progression and suppression [63]. Molecular studies have revealed cross-talk between necroptosis and p53/miRNA regulatory networks in oral cancer [7]. Furthermore, miR-140 has been shown to modulate PDGFRA–RIPK3/MLKL signaling, correlating with patient prognosis [64]. Preclinical work confirmed that the necroptosis inhibitor necrostatin-1 attenuates tumor growth and reprograms the immune microenvironment in OSCC xenografts [65]. In a 4-NQO-induced mouse model of OSCC, increased expression of necroptosis markers, including RIPK-1, RIPK-3, and MLKL, was observed in tumors and correlated with disease severity in human samples [65]. Treatment with the necroptosis inhibitor necrostatin-1 (NEC-1) reduces tumor progression, decreases the number and severity of lesions, and improves histology. Immune profiling revealed a shift towards an antitumor immune response, with reduced M2 macrophages, myeloid-derived suppressor cells, and increased M1 macrophages [65]. Shao et al. have reported that acetylshikonin induces necroptosis in human oral cancer cells by modulating mitochondrial function and oxidative stress-regulated signaling pathways via activating RIPK1, RIPK3, and MLKL [66]. Taken together, necroptosis plays a role in cancer, acting as both a tumor promoter and a therapeutic target. In OSCC, targeting necroptosis with inhibitors, such as NEC-1, or inducers, such as acetylshikonin, shows potential for modulating tumor progression and the tumor immune microenvironment, presenting promising avenues for therapeutic intervention. Table 4 summarizes therapeutic strategies targeting necroptosis in OSCC. This table outlines the key necroptosis-modulating agents, their mechanisms of action, primary molecular targets, and the observed therapeutic outcomes in OSCC models.

## 5. Targeting Pyroptosis in OSCC: Therapeutic Strategies and Prognostic Biomarkers

Pyroptosis is a type of programmed cell death driven by gasdermin (GSDM) activation and is characterized by excessive cell swelling and plasma membrane rupture, leading to the release of intracellular contents and triggering potent inflammatory and immune responses [67]. Upon activation by inflammasome-associated caspases (caspase-1/4/5/11), GSDMD forms pores in the plasma membrane, leading to cell lysis and the release of immune mediators. Figure 3B presents an overview of the key molecular mechanisms driving pyroptosis. Although inflammasome-activated pyroptosis primarily functions in immune defense, cancer-associated pyroptosis (CAP) involves other GSDMs and stimulates antitumor immunity. Despite sharing features such as membrane rupture, inflammasome-activated pyroptosis and CAP differ in their activation mechanisms and biological outcomes [68]. Chia et al. demonstrated that okanin, a natural compound derived from *Bidens pilosa* L., is effective against OSCC. Okanin demonstrated dose-dependent cytotoxicity against OSCC cell lines (SAS, SCC25, HSC3, and OEC-M1) by inducing G2/M cell cycle arrest and promoting both apoptosis and pyroptosis, as evidenced by increased caspase-3/7 activity and upregulation of pyroptosis markers such as caspase-1, GSDMC, GSDMD, and GSDME. In vivo, okanin significantly inhibited tumor growth in SAS xenograft models, with the treated tumors showing increased expression of pyroptosis-related markers and reduced tumor volume [69]. A study presents the development of Th-M, a mitochondria-targeted photosensitizer with aggregation-induced emission properties for type-I photodynamic therapy (PDT) in tongue squamous cell carcinoma (TSCC). Th-M effectively induces pyroptosis through mitochondrial dysfunction and subsequent cleavage of caspase-3 and GSDME under white light irradiation, leading to cancer cell death in vitro and tumor suppression in vivo. Additionally, Th-Ms exhibit a favorable biosafety profile [70]. Zi et al. reported that restoring GSDME expression in OSCC using the methyltransferase inhibitor decitabine enhanced chemotherapeutic sensitivity, induced pyroptosis, and modified the tumor microenvironment to improve immunotherapy outcomes [71]. In head and neck squamous cell carcinoma (HNSCC), GSDMB, GSDMD, and GSDME are upregulated in 57 tumors compared with 41 normal tissues and are associated with better survival. CTLA-4 blockade in 4MOSC1 models induces signal transducer and activator of transcription 1 (STAT1)/interferon regulatory factor 1 (IRF1)-dependent GSDMD/E activation via CD8^+^ T cell–derived interferon (IFN)-γ and TNF-α, an effect lost upon CD8^+^ depletion, highlighting gasdermin levels as a potential predictor of ICB response [72]. However, Xin et al. demonstrated the prognostic significance of pyroptosis-related long noncoding RNAs (lncRNAs) in OSCC [73]. Using RNA-seq data from The Cancer Genome Atlas (TCGA), eight pyroptosis-related lncRNAs (AC136475.2, AC024075.2, JPX, ZFAS1, TNFRSF10A-AS1, LINC00847, AC099850.3, and IER3-AS1) were identified to construct a prognostic signature that stratified OSCC patients into high- and low-risk groups. Kaplan–Meier survival curves demonstrate that patients classified as high-risk exhibit markedly poorer overall survival, while receiver operating characteristic (ROC) analysis confirms the signature’s predictive accuracy with an area under the curve (AUC) of 0.716. Immune infiltration profiling further indicates that these eight pyroptosis-related lncRNAs actively remodel the OSCC tumor microenvironment [73]. Targeting pyroptosis through natural compounds, photosensitizers, and epigenetic modulators shows promise for OSCC treatment by enhancing chemotherapeutic and immunotherapeutic responses. Pyroptosis-related lncRNAs also serve as potential prognostic biomarkers, providing novel insights into OSCC management (Table 5).

## 6. Targeting Ferroptosis in OSCC: Mechanisms, Prognostic Biomarkers, and Therapeutic Strategies

Ferroptosis is a distinct form of cell death triggered by iron-dependent phospholipid peroxidation and is regulated by various cellular metabolic pathways, including redox balance, iron metabolism, mitochondrial function, and the metabolism of amino acids, lipids, and sugars, along with multiple disease-related signaling pathways [74,75]. Ferroptosis can be triggered by iron ions and lipid components, such as polyunsaturated fatty acids, but can be suppressed by certain amino acid metabolites, such as glutathione (GSH). As a substrate of GPX4, GSH mitigates lipid peroxide toxicity (Figure 3C). However, when cells fail to neutralize excess ROS, accumulation of lipid peroxidation drives ferroptosis [76]. Therefore, ferroptosis acts as a tumor suppressor in OSCC development [77]. In our previous studies, we suggested that chrysophanol-based therapy could mitigate oral cancer progression through the activation of ferroptosis and modulate cell death, mTOR/peroxisome proliferator-activated receptor α (PPAR-α) signaling, and ROS accumulation in an iron-dependent manner [78,79]. Interestingly, plasma-activated Ringer’s lactate (PARL) solution, produced using a plasma-activated medium, contains reactive oxygen and nitrogen species (RONS) that can induce lipid peroxidation and ferroptosis in cancer cells. Researchers have demonstrated that PARL treatment leads to significant lipid peroxidation, glutathione depletion, and GPX4 inactivation in oral cancer cells, confirming that ferroptosis is the primary mechanism [80]. Wang et al. reported that a combination of melatonin and erastin effectively enhanced ROS-mediated apoptosis and ferroptosis in OSCC without significant side effects [81]. Trifluoperazine (TFP), an FDA-approved drug, increases lipid ROS levels and triggers ferroptotic cell death via autophagy activation, solute carrier family 7 member 11 (SLC7A11)/GPX4 axis inhibition, and mitochondrial damage. Molecular docking confirmed that TFP is a GPX4 inhibitor, and elevated GPX4 expression in oral cancer biopsies was associated with poor prognosis [82], suggesting that targeting ferroptosis can effectively suppress OSCC progression and represent a novel strategy for cancer therapy (Table 6).

A previous study established a prognostic model based on ferroptosis-related genes to predict overall survival in patients with OSCC patients. Using data from six datasets across three databases, the model identified nine key ferroptosis-related genes (*CISD2*, *DDIT4*, *CA9*, *ALOX15*, *ATG5*, *BECN1*, *BNIP3*, *PRDX5*, and *MAP1LC3A*) as prognostic markers. The model demonstrated high predictive accuracy, as validated by Kaplan–Meier analysis, ROC curves, and principal component analysis. Enrichment analysis revealed significant correlations between the risk score and the *TP53*-related, immune-related, and ferroptosis-related pathways. Further analysis revealed associations between risk scores, immune microenvironment characteristics, *TP53* mutations, and clinical treatment outcomes [83]. A study demonstrated that *PPT1* facilitates OSCC cell proliferation while suppressing ferroptosis [84]. *CDH4* expression was significantly higher in OSCC tissues than in normal tissues and correlated with poor patient survival. Functional assays demonstrated that *CDH4* promotes OSCC cell proliferation, migration, and invasion while reducing ferroptosis sensitivity. *CDH4* is positively associated with EMT pathway genes and ferroptosis suppressor genes and negatively associated with fatty acid metabolism and peroxisome pathway genes [85]. A comprehensive analysis of gene expression profiles and clinical data from publicly available databases showed that researchers identified the key ferroptosis-related genes (FRGs) associated with OSCC prognosis. Additionally, they examined the correlation between ferroptosis and immune cell infiltration, revealing that four FRGs (*FTH1*, *FLT3*, *CDKN2A*, and *DDIT3*) significantly influenced the tumor immune microenvironment [89]. Huang et al. reported that the *HOXA10*/*RUNX2* isoform II/*PRDX2* pathway mediates ferroptosis and apoptosis resistance in OSCC, promoting tumorigenesis [86]. Silencing *CK19* modulates the expression of *GPX4* and *ACSL4*, thereby regulating ferroptosis while simultaneously increasing MDA, Fe^2+^, and ROS levels, leading to the ferroptosis pathway activation in OSCC progression [87]. Previous findings suggested that quercetin exerts antitumor effects in OSCC by inducing ferroptosis through the mTOR/S6KP70 pathway [88]. In summary, multiple studies have identified key FRGs such as *CISD2, DDIT4*, and *CA9* as prognostic markers, demonstrating their association with tumor progression, immune microenvironment modulation, and treatment outcomes. Specific pathways, including the *HOXA10*/*RUNX2* isoform II/*PRDX2*, *CK19/GPX4/ACSL4*, and mTOR/S6KP70 pathways, have been shown to mediate ferroptosis resistance and tumor growth. Additionally, therapeutic agents such as quercetin, trifluoperazine, and PARL have been reported to enhance ferroptosis, suggesting that targeting ferroptosis may be a promising strategy for OSCC management. Table 6 presents key FRGs and pathways associated with prognosis and therapeutic targeting in OSCC. The table includes specific gene signatures, pathways, and molecular targets, highlighting their roles in modulating ferroptosis sensitivity, tumor proliferation, and treatment outcomes in OSCC, suggesting that the integration of ferroptosis-based strategies may provide novel therapeutic avenues for treating OSCC.

## 7. Regulated Cell Death Pathways as Therapeutic Targets in OSCC: Apoptosis, ACD, Necroptosis, Pyroptosis, and Ferroptosis

Therapeutic strategies targeting cell death pathways in oral cancer are rapidly evolving as promising approaches to improve treatment outcomes [7]. By understanding the distinct molecular mechanisms governing apoptosis, ACD, necroptosis, pyroptosis, and ferroptosis, researchers can develop targeted therapies that effectively eliminate cancer cells while minimizing their toxicity to normal tissues.

Therapeutic agents that restore apoptotic signaling in OSCC include BH3 mimetics such as venetoclax [90], death receptor agonists such as TRAIL [91], and p53 reactivators such as APR-246 [24]. BH3 mimetics can selectively inhibit anti-apoptotic proteins such as Bcl-2, thus restoring the balance between pro- and anti-apoptotic factors [92]. Moreover, TRAIL receptor agonists have been shown to induce apoptosis in OSCC cells while sparing normal cell types [91]. In addition, Mortezagholi et al. demonstrated that miR-34 expression is downregulated in paclitaxel-resistant OECM-1/PTX cells. Restoration of miR-34 increases paclitaxel sensitivity by reducing P-glycoprotein (P-gp) levels and enhancing DNA damage and apoptosis through the upregulation of the p53/ataxia-telangiectasia mutated (ATM)/ATM and Rad3-related (ATR)/checkpoint kinase (CHK) 1/CHK2 pathway [93]. Given the critical role of ACD in OSCC, therapeutic targeting is nuanced [8]. Autophagy inhibitors, such as CQ and hydroxychloroquine, are currently being investigated in clinical trials to prevent cancer cells from using autophagy as a survival mechanism under therapeutic stress [94]. Rapamycin has been shown to inhibit the growth of oral cancer cells through mechanisms that include the induction of ACD, the generation of oxidative stress, and the inhibition of key signaling pathways such as extracellular signal-regulated kinase 1 and 2 (ERK1/2), nuclear factor kappa-light-chain-enhancer of activated B cells (NF-κB), and β-catenin [95]. Small molecules targeting RIPK1, RIPK3, and MLKL can selectively trigger necroptosis in OSCC [63]. Qi and Tang explored the role of pyroptosis-related genes in OSCC using data from TCGA. A prognostic model based on three key genes, cytotoxic T-lymphocyte-associated protein 4 (*CTLA4*), cluster of differentiation 5 (*CD5*), and interleukin-12 receptor subunit beta-2 (*IL12RB2*), was developed, and the high expression of these genes was associated with a lower risk score and improved overall survival. Additionally, leukocyte-associated immunoglobulin-like receptor 2 (*LAIR2*) was identified as a hub gene that was negatively correlated with the risk score and immune cell infiltration [96]. Zeng et al. have successfully developed a pyroptosis-related prognostic signature that may serve as a potential predictor of OSCC outcomes. These findings suggest that pyroptosis-related regulators are promising biomarkers for OSCC diagnosis and treatment [97]. Targeting ferroptosis holds the potential for treating OSCC with high oxidative stress and iron metabolism dysregulation [98]. Compounds such as erastin and RSL3, which inhibit GPX4 and promote lipid peroxidation, have been shown to effectively induce ferroptosis in HNSCC, including OSCC [99,100]. Therefore, targeting multiple regulated cell death pathways, including apoptosis, ACD, necroptosis, pyroptosis, and ferroptosis, represents a comprehensive approach to OSCC treatment. Therapeutic agents that modulate these pathways are potential predictive biomarkers and treatment targets for improving OSCC outcomes. Table 7 summarizes therapeutic strategies targeting regulated cell death pathways in OSCC. The table includes information on apoptosis, ACD, necroptosis, pyroptosis, and ferroptosis, along with key therapeutic agents, mechanisms of action, molecular targets, and observed outcomes in OSCC models. These approaches provide potential treatment strategies to overcome drug resistance and enhance the management of OSCC.

## 8. Disulfidptosis in SLC7A11-High OSCC: Mechanisms, Therapeutic Implications, and Prognostic Signatures

Disulfidptosis is a newly identified form of cell death induced by disulfide stress, marked by the collapse of cytoskeletal proteins and F-actin as a result of intracellular disulfide accumulation, and was reported by Liu et al. They demonstrated that disulfidptosis occurs in SLC7A11-high cancer cells under glucose starvation [101]. Unlike apoptosis or ferroptosis, disulfidptosis is driven by the aberrant accumulation of intracellular disulfides, leading to disulfide bonding in actin cytoskeleton proteins and F-actin collapse. The process is dependent on SLC7A11-mediated cystine uptake and modulated by the WAVE regulatory complex and Rac signaling. Importantly, glucose transporter inhibitors can induce disulfidptosis and suppress tumor growth in SLC7A11-high cancers [102]. In SLC7A11-high cancer cells, glucose deprivation leads to NADPH depletion and abnormal accumulation of disulfides, promoting disulfide bonding within actin cytoskeletal proteins. This disrupts the actin network and triggers disulfidptosis, a regulated form of cell death driven by disulfide stress [103]. The study establishes and validates a novel prognostic signature based on disulfidptosis-related long non-coding RNAs in patients with HPV-negative OSCC [104]. Wu et al. also developed a novel prognostic risk model for OSCC based on disulfidptosis-related immune genes (DRIGs). This DRIG-based risk model provides a valuable tool for predicting OSCC prognosis and offers insights into the tumor immune microenvironment and immunotherapy responsiveness, supporting its potential application in personalized cancer management [105].

## 9. Summary

OSCC remains a significant health burden owing to its aggressive nature and poor prognosis. Targeting regulated cell death pathways, such as apoptosis, ACD, necroptosis, pyroptosis, and ferroptosis, has emerged as a promising therapeutic strategy. Apoptosis can be restored using BH3 mimetics, TRAIL receptor agonists, and p53 reactivators, whereas ACD modulation plays a key role in tumor suppression and survival. Necroptosis and pyroptosis can also be leveraged to induce cancer cell death using the potential biomarkers and therapeutic agents identified in recent studies. Ferroptosis driven by iron-dependent lipid peroxidation has gained attention as a therapeutic target, with compounds such as quercetin, trifluoperazine, and PARL showing efficacy in preclinical models. Overall, integrating these cell death pathways into OSCC treatment may improve the therapeutic outcomes and provide novel prognostic biomarkers.

Table 8 compiles recent findings on regulated cell death (RCD) mechanisms in OSCC, including apoptosis, ACD, necroptosis, pyroptosis, ferroptosis, and disulfidptosis. For each entry, the specific gene, protein, or lncRNA involved is listed, along with the associated cell death pathway, any known therapeutic modulator or compound, the experimental model used (in vitro, in vivo, or clinical data), and the source of the evidence. The table highlights both established and emerging targets with relevance to OSCC pathogenesis and therapy, providing a foundation for future translational research and drug development.

## 10. Limitations

While this review aims to provide a comprehensive overview of regulated cell death pathways and their therapeutic relevance in OSCC, several limitations should be acknowledged. First, although every effort was made to focus on OSCC-specific literature, in some sections—particularly those discussing apoptosis and ACD—references were drawn from broader HNSCC or other tumor types due to the limited availability of OSCC-exclusive studies on certain mechanisms. Given the molecular heterogeneity among HNSCC subtypes, we recognize that extrapolation from other cancers may not fully capture the unique biological behavior of OSCC. Additionally, some of the initial citations relied on review articles rather than primary experimental data. This has been addressed in the revised version by incorporating more recent and OSCC-specific original research; however, future work should continue to refine our understanding with high-quality, tumor-specific studies. These limitations underscore the need for continued investigation into OSCC-specific mechanisms of cell death and their therapeutic implications.

## 11. Future Perspectives

An integrative therapeutic strategy that simultaneously engages apoptosis, ACD, necroptosis, pyroptosis, and ferroptosis in OSCC could, in principle, yield more complete tumor eradication than single-pathway approaches, as recent comprehensive analyses have underscored the complex interplay and potential synergy among these regulated cell death modalities in oral cancer. Combining immunotherapy with targeted modulators (inducers or inhibitors) of ACD, ferroptosis, pyroptosis, and necroptosis may elicit robust antitumor activity even in tumors resistant to immune checkpoint inhibitors [7,106]. By activating complementary death mechanisms, including mitochondrial cytochrome-c release and caspase cascade activation (apoptosis), autophagic vesicle formation and lysosomal degradation (ACD), RIPK3/MLKL-mediated membrane permeabilization (necroptosis), gasdermin-driven pore formation (pyroptosis), and iron-catalyzed lipid peroxidation (ferroptosis), this multipronged regimen may reduce the likelihood of residual malignant cells escaping through blockade or mutation of any single pathway, thereby delaying or preventing the emergence of resistant clones. Indeed, several preclinical studies have demonstrated that ferroptosis inducers such as erastin decrease OSCC cell viability by approximately 50% in vitro and suppress xenograft tumor volume by over 50% in vivo [99].

Nonetheless, concurrent induction of multiple regulated cell death pathways raises significant safety concerns. Combination of non-apoptotic RCD modulators with immunotherapy in murine models has produced synergistic tumor regression but also elicited marked increases in systemic cytokines (e.g., IL-1β and IL-6) and transient liver enzyme elevations, indicating a risk of off-target inflammatory toxicity akin to a cytokine storm [107]. Normal tissues exhibit heterogeneous sensitivity to each death modality, and without sufficiently selective delivery, off-tumor toxicity could manifest as damage to high-turnover epithelia or critical organ systems. Furthermore, the pharmacological combination of pathway modulators increases the risk of unanticipated drug–drug and drug–protein interactions, potentially exacerbating oxidative stress and systemic inflammation.

To address these challenges, future investigations should prioritize the development of precision delivery platforms, for example, iron-oxide nanoparticle systems capable of co-delivering ferroptosis inducers and chemotherapeutics that have been shown to reverse chemoresistance while confining activity to tumor tissue in preclinical models [108], and should incorporate real-time biomarker assays (e.g., circulating lipid peroxides and gasdermin-cleavage fragments) to monitor pathway engagement and guide adaptive dosing. Rigorous validation across heterogeneous preclinical OSCC models that reflect the full spectrum of clinical stages and molecular subtypes will be essential to optimize the balance between enhanced tumor cell kill and acceptable toxicity prior to clinical translation.

## 12. Conclusions

In this review, we have explored how five regulated cell death pathways, apoptosis, ACD, necroptosis, pyroptosis, and ferroptosis, contribute to OSCC development and therapy. By restoring apoptotic signaling, fine-tuning autophagic flux, triggering inflammatory death via necroptosis and pyroptosis and inducing iron-dependent lipid peroxidation, each modality presents a unique therapeutic opportunity. When these mechanisms are combined within a single treatment framework alongside immune checkpoint inhibitors, they can overcome single-pathway resistance and achieve more durable tumor control. However, the synergistic promise of a multimodal cell death approach must be carefully balanced against potential safety risks.

## Figures and Tables

**Table 1:** Therapeutic Strategies Targeting Apoptosis in OSCC.

Therapeutic Strategy	Target	Cancer Cell Type/Model	Key Findings	References
IAP overexpression (e.g., XIAP and survivin)	IAP family proteins	OSCC tissues and cell lines (e.g., SCC-9 and CAL-27)	Overexpression linked to resistance to apoptosis, poor prognosis	[20,21]
BH3 mimetics (e.g., ABT-199/Venetoclax)	Bcl-2 family proteins	OSCC cell lines (e.g., HSC-3 and SCC-25)	Induced intrinsic apoptosis, enhanced sensitivity to chemotherapy	[23]
p53 reactivation (e.g., PRIMA-1 and APR-246)	Mutant *p53*	OSCC models with *p53* mutation	Restored *p53* function, induced apoptosis	[24]
Death receptor agonists (e.g., TRAIL)	TRAIL receptors (DR4/DR5)	OSCC xenograft models, CAL-27, SCC-15	Triggered extrinsic apoptosis, though variable efficacy due to resistance	[25]
SMAC mimetics (e.g., LCL161 and BV6)	IAP antagonism	OSCC cell lines and preclinical mouse models	Sensitized cells to apoptosis, especially when combined with TRAIL or chemo	[26]
Combination therapies (e.g., with ICIs)	Multiple (apoptosis + immune checkpoint)	Syngeneic or humanized OSCC mouse models	Enhanced tumor regression and immune activation	[27,28]

**Table 2:** Therapeutic Strategies Targeting Autophagy in OSCC and Other Cancers.

Cancer Type	Experimental Model	Autophagy Marker(s)	Findings	References
OSCC	Human tissue samples and cell lines (e.g., SCC-9 and HSC-3)	Beclin-1, LC3	Loss of Beclin-1 and reduced LC3 expression correlated with tumor progression and poor prognosis, suggesting a tumor-suppressive role of autophagy.	[19]
HNSCC	Genomic and transcriptomic analysis of patient cohorts	NKX2-3, FADD	Identified as autophagy-related genes linked to poor prognosis and potential therapeutic targeting.	[46]
HNSCC	Bioinformatics and IHC validation	PARK2, EGFR	Involved in autophagy regulation; PARK2 acts as a tumor suppressor, while EGFR is implicated in autophagy-mediated survival signaling.	[47]
OSCC	In vitro cell culture under stress (hypoxia, nutrient deprivation)	LC3-II, p62	Autophagy confers survival advantage under metabolic stress and may mediate resistance to therapy.	[48]
OSCC and other solid tumors	Preclinical studies using autophagy modulators	ROS, LC3, p62	ROS both trigger and result from autophagy. Pathways such as PI3K/AKT/mTOR and AMPK regulate this loop, offering therapeutic targets.	[45,49]

**Table 3:** Therapeutic Approaches Targeting ACD in OSCC.

Therapeutic Agent(s)	Cancer Cell Type	Key Mechanisms Targeted	Experimental Model	References
NaAsO_2_ + CQ + DCA	OSCC cells	Oxidative stress, ACD, glycolysis	In vivo (Xenograft mouse model)	[51]
Apicidin	OSCC cells	Apoptosis and ACD	In vitro	[52]
SPD	OSCC cells	ACD induction, reduced DNA damage and oxidative stress	In vivo (4NQO-induced mouse oral carcinogenesis model)	[53]
Resveratrol	Cisplatin-resistant oral cancer CAR cells	Mitochondria-dependent apoptosis and ACD via AMPK activation	In vitro	[54]
Piperine	Oral cancer cells	Apoptosis and autophagy via PI3K/AKT/mTOR inhibition	In vitro and in vivo (Xenograft mouse model)	[55]
Bortezomib	OSCC cells	Radiosensitization via autophagic degradation of TRAF6	In vitro and in vivo (Xenograft mouse model)	[56]

**Table 4:** Therapeutic Strategies Targeting Necroptosis in OSCC.

Model Type	Cell/Cancer Type	Key Findings	References
In vitro and Ex vivo	OSCC cell lines and patient data	miR-140 modulates PDGFRA–RIPK3/MLKL signaling; expression correlates with patient prognosis.	[64]
Ex vivo	OSCC patient samples	RIPK1, RIPK3, and MLKL expression correlates with disease severity; necroptosis implicated in OSCC progression.	[65]
In vivo	4-NQO-induced mouse oral carcinogenesis model	Increased expression of necroptosis markers (e.g., RIPK1, RIPK3, and MLKL) correlates with tumor severity; NEC-1 treatment reduces tumor burden and improves histopathology.	[65]
In vivo	OSCC xenograft mouse model	NEC-1 reduces tumor growth and reshapes the immune microenvironment (↓M2 macrophages, ↓myeloid-derived suppressor cells, ↑M1 macrophages).	[65]
In vitro	Human OSCC cell lines	Acetylshikonin induces necroptosis by activating RIPK1, RIPK3, and MLKL via mitochondrial and oxidative stress-regulated pathways.	[66]

**Table 5:** Therapeutic Strategies Targeting Pyroptosis in OSCC.

Cancer Cell Type/Model	Experimental Model	Mechanism/Outcome	Reference
OSCC cell lines: SAS, SCC25, HSC3, OEC-M1	In vitro; in vivo (SAS xenograft mouse model)	Okanin induced dose-dependent cytotoxicity via G2/M arrest, apoptosis, and pyroptosis (↑CASP1, GSDMC/D/E, caspase-3/7); reduced tumor volume in vivo.	[69]
TSCC cells	In vitro (Light-irradiated Th-M); in vivo (Tumor-bearing mice)	Th-M induced mitochondrial dysfunction, leading to caspase-3 and GSDME–mediated pyroptosis; effective tumor suppression with favorable biosafety.	[70]
OSCC cells (unspecified lines)	In vitro; preclinical immunotherapy setting	Decitabine restored GSDME expression, enhancing pyroptosis and chemotherapy response; improved immune microenvironment and ICB response.	[71]
HNSCC tumor samples (*n* = 57), normal tissues (*n* = 41); HNSCC cell line: 4MOSC1	Ex vivo (Human tissue); in vivo (4MOSC1 orthotopic mouse model)	GSDMB/D/E upregulated in tumors; CTLA-4 blockade activated GSDMD/E via IFN-γ/TNF-α signaling (STAT1/IRF1 axis); effect CD8^+^ T cell–dependent.	[72]
OSCC (TCGA dataset)	Bioinformatic analysis (RNA-seq)	Identified 8 pyroptosis-related lncRNAs predictive of survival (AUC = 0.716); lncRNAs modulated immune infiltration and OSCC microenvironment.	[73]

↑, Up-regulation; n, sample size.

**Table 6:** Preclinical Combination Therapies Engaging Multiple Regulated Cell Death Pathways in OSCC.

Therapeutic Strategy	Modulator and Molecular Axis	Mechanism of Action	Key Targets	Outcomes in OSCC	References
Agent	Chrysophanol	Activates ferroptosis, modulates mTOR/PPAR-α signaling, ROS accumulation	mTOR, PPAR-α, ROS	Reduces tumor progression via iron-dependent cell death	[78,79]
	PARL	Induces lipid peroxidation, glutathione depletion, GPX4 inactivation	RONS, GPX4, lipid ROS	Increased ferroptosis, reduced cancer cell viability	[80]
	Melatonin + Erastin	Enhances ROS-mediated apoptosis and ferroptosis	ROS, lipid peroxides	Promotes ferroptosis with minimal side effects	[81]
	TFP	Increases lipid ROS, inhibits SLC7A11/GPX4 axis, activates autophagy	SLC7A11, GPX4, lipid ROS	Induces ferroptosis, reduces mitochondrial function, poor prognosis in OSCC	[82]
Gene/Pathway	Ferroptosis-Related Gene Signature	Predicts overall survival, correlates with *TP53*, immune response	CDGSH iron sulfur domain 2 (*CISD2*), DNA damage inducible transcript 4 (*DDIT4*), carbonic anhydrase 9 (*CA9*), arachidonate 15-lipoxygenase (*ALOX15*), *ATG5*, *BECN1*, BCL2/adenovirus E1B 19 kDa protein-interacting protein 3 (*BNIP3*), peroxiredoxin 5 (*PRDX5*), microtubule-associated proteins 1A/1B light chain 3A (*MAP1LC3A*)	High predictive accuracy for OS in OSCC, associated with tumor progression	[83]
	Palmitoyl-protein thioesterase 1 (*PPT1*)	Suppresses ferroptosis, promotes OSCC proliferation	*PPT1*	Enhances tumor growth, poor prognosis	[84]
	Cadherin-4 (*CDH4*)	Reduces ferroptosis sensitivity, promotes proliferation and invasion	*CDH4*, epithelial–mesenchymal transition (EMT)-related genes	Correlation with poor survival, increased EMT and ferroptosis resistance	[85]
	Homeobox A10 (*HOXA10*)/RUNT-related transcription factor 2 (*RUNX2*) isoform II/peroxiredoxin-2 (*PRDX2*) Axis	Inhibits ferroptosis and apoptosis, promotes tumorigenesis	*HOXA10*, *RUNX2* isoform II, *PRDX2*	Ferroptosis resistance, tumor progression	[86]
	Cytokeratin 19 (*CK19*)/*GPX4*/acyl-CoA synthetase long chain family member 4 (*ACSL4*) Axis	Regulates ferroptosis, increases ROS and lipid peroxidation	*GPX4*, *ACSL4*, malondialdehyde (MDA), Fe^2+^	Activation of ferroptosis pathway, increased ROS accumulation	[87]
	mTOR/S6KP70 Pathway	Induces ferroptosis via ROS accumulation	mTOR, S6KP70	Quercetin-mediated ferroptosis induction, tumor suppression	[88]

**Table 7:** Therapeutic Strategies Targeting Regulated Cell Death Pathways in OSCC.

Cell Death Pathway	Therapeutic Agent/Strategy	Mechanism of Action	Key Targets	Outcomes in OSCC	References
Apoptosis	p53 Reactivators (APR-246)	Restores p53 function, induces apoptosis	p53, ATM/ATR, CHK1/CHK2	Increased chemosensitivity, enhanced apoptosis	[24]
	BH3 Mimetics (Venetoclax)	Inhibits anti-apoptotic Bcl-2 proteins, restores apoptotic signaling	Bcl-2, Bax/Bak	Enhanced apoptosis, reduced tumor growth	[90,92]
	Death Receptor Agonists (TRAIL)	Activates extrinsic apoptotic pathway	TRAIL-R, Fas, TNFR1	Selective apoptosis induction in OSCC cells	[91]
	miR-34 Restoration	Reduces P-gp expression, enhances DNA damage and apoptosis	P-gp, p53/ATM/ATR pathway	Reverses paclitaxel resistance, promotes apoptosis	[93]
Autophagy	CQ, HCQ	Inhibits autophagosome–lysosome fusion	LC3, BECN1, p62	Prevents autophagy-mediated survival under therapeutic stress	[94]
ACD	Rapamycin	Induces ACD, oxidative stress, inhibits signaling pathways	ERK1/2, NF-κB, β-catenin	Suppresses OSCC growth, promotes cell death	[95]
Necroptosis	Small Molecules (RIPK1, RIPK3, MLKL Inhibitors)	Induces necroptosis via death receptor pathways	RIPK1, RIPK3, MLKL	Promotes necroptosis, reduces tumor viability	[63]
Pyroptosis	CTLA-4 Blockade	Induces gasdermin-mediated pyroptosis via immune activation	CTLA-4, GSDMs	Enhances immune-mediated tumor cell death	[96]
	Pyroptosis-related lncRNAs	Prognostic signature for risk stratification	CTLA4, CD5, IL12RB2	Predicts patient survival, modulates immune response	[97]
Ferroptosis	Erastin, RSL3	Inhibits GPX4, promotes lipid peroxidation	GPX4, lipid ROS	Induces ferroptosis, reduces OSCC cell viability	[99,100]

**Table 8:** Summary of Key Genes, Pathways, Therapeutic Agents, and Experimental Models Targeting Regulated Cell Death in OSCC.

Gene or Protein or lncRNA	Cell Death Pathway	Therapeutic Agent/Modulator	Experimental Model	References
Bcl-2/Bax/Bcl-xL	Apoptosis	BH3 mimetics (ABT-199/Venetoclax)	OSCC cell lines	[23,92]
p53	Apoptosis	APR-246, PRIMA-1	OSCC cell lines, clinical data	[24]
TRAIL-R/Fas/TNFR1	Apoptosis	TRAIL receptor agonists	OSCC cell lines	[25]
NKX2-3, FADD, PARK2	Autophagy	-	OSCC patient data	[46]
BECN1, LC3, p62	ACD	Chloroquine, Rapamycin	OSCC cell lines, mouse model	[48,95]
RIPK1/RIPK3/MLKL	Necroptosis	Necrostatin-1 (NEC-1)	4NQO-induced OSCC mouse model	[65]
Acetylshikonin	Necroptosis	Natural compound	Human OSCC cell lines	[66]
CASP1, GSDMD, GSDME	Pyroptosis	Okanin, Decitabine, Th-M	SAS xenograft, OSCC cell lines	[69,70,71]
Pyroptosis-related lncRNAs	Pyroptosis	-	TCGA OSCC datasets	[73]
GPX4, SLC7A11	Ferroptosis	Erastin, RSL3, Trifluoperazine (TFP)	OSCC cell lines, patient tissues	[78,79,80,81,82]
*HOXA10*/*RUNX2*/*PRDX2*	Ferroptosis	-	OSCC cell lines, patient tissue analysis	[86]
*CK19*, *ACSL4*	Ferroptosis	-	OSCC cell lines	[87]
Quercetin	Ferroptosis	Natural flavonoid	OSCC cell lines	[88]
SLC7A11	Disulfidptosis	Glucose transporter inhibitors	SLC7A11-high OSCC models	[102]
Disulfidptosis-related lncRNAs	Disulfidptosis	-	HPV-negative OSCC datasets, cell lines	[104]

## Data Availability

Not applicable.

## Abbreviations

ACD	Autophagic Cell Death
ACSL4	Acyl-CoA synthetase long chain family member 4
AIF	Apoptosis-inducing factor
AKT	Protein Kinase B
AMPK	AMP-activated Protein Kinase
Apaf-1	Apoptotic Protease-activating Factor 1
ATG	Autophagy-related
ATM	Ataxia-telangiectasia mutated
ATP	Adenosine triphosphate
AUC	Area under the curve
Bak	Bcl-2 homologous antagonist/killer
Bax	Bcl2-associated X protein
BECN1	Beclin-1
Bcl-2	B-cell lymphoma 2
Bcl-xL	B-cell lymphoma-extra large
BID	BH3 interacting-domain death agonist
BNIP3	BCL2/adenovirus E1B 19 kDa protein-interacting protein 3
CA9	Carbonic anhydrase 9
CAP	Cancer-associated pyroptosis
CAR	Chimeric antigen receptor
Caspase	Cysteine-dependent aspartate-specific protease
CD5	Cluster of differentiation 5
CHK	Checkpoint kinase
CISD2	CDGSH iron sulfur domain 2
CK19	Cytokeratin 19
CQ	Chloroquine
CTLA4	Cytotoxic T-lymphocyte-associated protein 4
DAMPs	Damage-associated molecular patterns
DCA	Dichloroacetate
DDIT4	DNA damage inducible transcript 4
DISC	Death-inducing Signaling Complex
DRIGs	Disulfidptosis-related Immune Genes
DRs	Death Receptors
EGFR	Epidermal Growth Factor Receptor
EMT	Epithelial–mesenchymal Transition
Endo G	Endonuclease G
ER	Endoplasmic Reticulum
ERK1/2	Extracellular signal-regulated kinase 1 and 2
FADD	Fas-associated Death Domain Protein
Fas	Fas cell surface death receptor
FRGs	Ferroptosis-related Genes
GPX4	Glutathione Peroxidase 4
GSDM	Gasdermin
GSDMD	Gasdermin D
GSH	Glutathione
GTPase	Guanosine triphosphatase
HCQ	Hydroxychloroquine
HDAC	Histone Deacetylase
HNSCC	Head and Neck Squamous Cell Carcinoma
HOXA10	Homeobox A10
HOPS	Homotypic fusion and vacuolar protein sorting
IAP	Inhibitor of Apoptosis Protein
IFN	Interferon
IL	Interleukin
IL12RB2	Interleukin-12 receptor subunit beta-2
IRF1	Interferon regulatory factor 1
LAIR2	Leukocyte-associated immunoglobulin-like receptor 2
LC3	Microtubule-associated Protein 1A/1B-light Chain 3
LDH	Lactate dehydrogenase
lncRNAs	Long Noncoding RNAs
LPS	Lipopolysaccharide
MAP1LC3A	Microtubule-associated proteins 1A/1B light chain 3A
MDA	Malondialdehyde
MLKL	Mixed Lineage Kinase Domain-like Pseudokinase
MOMP	Mitochondrial Outer Membrane Permeabilization
mTOR	Mammalian Target of Rapamycin
NEC-1	Necrostatin-1
NF-κB	Nuclear factor kappa-light-chain-enhancer of activated B cells
NKX2-3	NK2 Homeobox 3
OSCC	Oral Squamous Cell Carcinoma
P-gp	P-glycoprotein
PARK2	Parkin RBR E3 ubiquitin protein ligase
PARL	Plasma-activated Ringer’s Lactate
PARP1	Poly (ADP-ribose) Polymerase 1
PE	Phosphatidylethanolamine
PDT	Photodynamic Therapy
PI3K	Phosphoinositide 3-kinase
PPAR-α	Peroxisome proliferator activated receptor α
PPT1	Palmitoyl-protein Thioesterase 1
PRDX2	Peroxiredoxin-2
PRDX5	Peroxiredoxin 5
Rab7	Ras-related protein 7
RCD	Regulated Cell Death
RIG-I	Retinoic acid-inducible gene I
RIPK1/3	Receptor-interacting Protein Kinases 1 and 3
ROC	Receiver operating characteristic
RONS	Reactive Oxygen and Nitrogen Species
ROS	Reactive Oxygen Species
RUNX2	RUNT-related Transcription Factor 2
SL7A11	Solute carrier family 7 member 11
SMAC	Second Mitochondria-derived Activator of Caspases
SNARE	Soluble N-ethylmaleimide-sensitive factor activating protein receptor
SPD	Spermidine
SQSTM1	Sequestosome 1
STAT1	Signal transducer and activator of transcription 1
STING	Stimulator of interferon genes
TCGA	The Cancer Genome Atlas
TFP	Trifluoperazine
TICAM1	TIR domain containing adaptor molecule 1
TLRs	Toll-like receptors
TNF	Tumor Necrosis Factor
TP53	Tumor protein p53
TRAF6	Tumor necrosis factor receptor-associated factor 6
TRAIL	TNF-related Apoptosis-inducing Ligand
TRAIL-R	TNF-related apoptosis-inducing ligand receptor
TSCC	Tongue Squamous Cell Carcinoma
ULK1	Unc-51-like Kinase 1
VPS34	Vacuolar protein sorting 34
XIAP	X-linked IAP
ZBP1	Z-DNA binding protein 1
